# Incremental Construction for Scalable Component-Based Systems

**DOI:** 10.3390/s20051435

**Published:** 2020-03-06

**Authors:** Tauseef Rana, Abdullah Baz

**Affiliations:** 1 Department of Computer Software Engineering, MCS, National University of Sciences and Technology, Islamabad 44000, Pakistan; 2 Department of Computer Engineering, College of Computer and Information Systems, Umm Al-Qura University, Makkah P.O.Box 715, Saudi Arabia; aobaz01@uqu.edu.sa

**Keywords:** incremental construction, EX-MAN, ADLs, web-services, composition, component model, exogenous connector

## Abstract

The availability of smart and intelligent sensors has changed the monitoring, control and maintenance of a conventional and advanced cyber-physical system used in public or private sectors of a society. For example, internet of things (IoT)-based health, agricultural and weather management systems. With the emergence of such sensors, along with the new ways to communicate or coordinate with them, we need to analyze and optimize the system construction processes. In this paper, to address the issue of scalability for bigger and complex systems based on sensors, we redefine an incremental construction process with an emphasis on behavior preservation and study the effectiveness of the use of software component models from the component-based development domain. In this paper, to deal with the issue of scalability, we investigate component-based development approaches with respect to our defined process and propose a taxonomy of component models with respect to component/system behavior. Moreover, based on the outcome of our analysis, we recommend the EX-MAN component model as the most suitable approach. We investigate incremental construction in the context of the three main categories of current component models, namely models where components are: (i) objects, (ii) architectural units and (iii) encapsulated components. Furthermore, to evaluate our defined process and selection of EX-MAN, we designed three examples of systems using our proposed process in EX-MAN component model.

## 1. Introduction

Technological advancements have made many dreamed autonomous and intelligent systems possible these days. The role of smart sensors [1] for the construction of a cyber-physical system (CPS) is vital. Based on the internet of things (IoT) [2,3], many companies offer services to quickly make such systems. Some of these systems are critical systems [4,5]. Keeping in view the aforementioned, we have entered in the era where bigger and complex systems are created from existing heterogeneous subsystems. In this context, for the CPS domain, the open issues may include scalability, heterogeneity, distribution, real-time optimization, self-adaptability and etc. The scope of this paper is to work on the scalability issue as the construction of bigger and complex systems in a shorter time is more needed than ever.

In general, there are two kinds of software based systems: systems with changeable requirements and systems with relatively fixed requirements. The system specifications driven by external factors (e.g., government policies) are best built by following agile methods [6]. However, a CPS is more stable for its specifications and such a system can be best built by a more bureaucratic process models (e.g., waterfall model [7]). In constructing a CPS, coping with the scale and ever-increasing complexity in manageable incremental and iterative steps is highly desired.

The concept of an incremental and iterative process to construct systems is very old [8,9]. In practice, many system development/construction processes deal with incompleteness or imprecision in system requirements. For example, in an incremental step, an existing system behavior may be changed or deleted in the agile and evolutionary software development methods [6,10]. Hence, the natural benefit of such a construction process is its ability to incorporate the change in the system requirement; however, an incremental step does not mean adding new functionality.

Incremental system construction aims to build systems iteratively by adding increments in a stepwise manner, starting from a small initial system. Such an approach holds the promise of managing scale and complexity, and should therefore be useful for building large systems. In the context of software development, many approaches based on refinement have been proposed for incremental system construction. In general, these approaches are top-down and not bottom-up. The view taken in this paper is that incremental construction is intrinsically bottom-up and would therefore be easier to achieve by means of component-based development (CBD), which is also intrinsically bottom-up.

In this paper, based on the behavior containment and preservation, we define our notion of incremental construction by which we focus only on growing the system in increments, i.e., incremental construction. In our view, an iterative software construction approach is incremental if the functionality of the system under construction is incremented in each successive iteration. This means that in the construction process functionalities are only added, and not altered or deleted. Such an approach is applicable to construct systems with fixed requirements or specifications.

This paper contributes a redefinition of the notion of incremental construction with an emphasis on the behavior containment. Next, in the context of incremental construction, we contribute a study of current component models to investigate how incremental construction can be achieved in the three main categories of current component models, namely models where components are: (i) objects, (ii) architectural units, and (iii) encapsulated components. This study has not been presented in the literature before. By studying and comparing the strengths and weaknesses of component models from the three categories, we propose a taxonomy of component models with respect to component/system behavior and select the EX-MAN component model (EX-MAN) [11,12] for its comparative suitability for incremental construction. For the evaluation of our scalable approach, using EX-MAN, we constructed and tested many systems. In this paper, we discuss three such system construction.

The rest of the paper is organized into six sections. Section 2 presents the salient approaches known for incremental and iterative development; moreover, the CBD domain is introduced here. In Section 3, we introduce CBD by different kinds of components and compositions; the contribution here is a taxonomy of composition mechanisms in CBD. Moreover, we redefine the notion of incremental construction based on behavior containment for CBD in this section. Furthermore, this section outlines the methodology of our study in this paper. Next, in Section 4, we analyze achieving incremental construction in the three categories of component models. Furthermore, this section contributes a taxonomy of component models with respect to component/system behavior. Using the best component model EX-MAN, we present the few system designs in Section 5. Section 6 includes discussion and Section 7 presents the conclusion and set directions for future work.

## 2. Related Work

Developing a program/software in steps by following an incremental and iterative process is an old and fundamental approach [8,13,14]. Primarily, software development/construction approaches are either a top-down approach or a bottom-up approach. In top-down program development approaches, a top level abstract program with intended functional interface (and no code) is created as an initial step. As a consequence, these approaches do not scale up in general. In our view, CBD can help to improve the situation. In CBD, system construction is bottom-up. In contrast to the top-down construction approaches, in bottom-up approaches, program units are composed to yield a larger program unit. For system construction in a bottom-up (or synthesis) approach, software composition plays a key role.

In a published article in year 2003, Larman and Basili highlight the concept of incremental and iterative development (IID) process to construct systems in the historical perspective [9]. These approaches allow software construction in steps; in a step, the under construction system gets new features or existing features may be altered or deleted. Our proposed approach is for CBD; hence, in this context, incremental approaches and CBD are our closely related work areas.

In CBD, in a system, interactions between the components is an important element that can help in finding the ways of composing components together [15]. For CPS construction, CBD-based development, by using and reusing secure components [16,17], is in need of the latest development methodologies. These systems may be comprised of many soft and hard components distributed in different locations [18]. In the aforesaid context, the importance of interfaces of components becomes very important.

**Figure 1:**
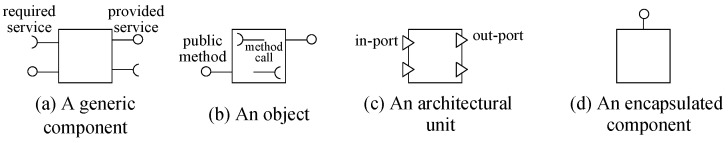
Components in current component models.

Reuse of existing work in software engineering is highly demanded to reduce the development cost and to gain high quality software [19]. In CBD [20,21], basic and composite components are developed for reuse and composite components and systems are created by reuse [22]. A generic component in CBD (Figure 1a) can be represented by a component diagram from UML. Components in CBD can be divided into three groups (objects, architectural units and encapsulated components) based on the interfaces of components [15].

An object (Figure 1b) has public methods (correspond to the provided services of the generic component) and external method calls (corresponding to the required services of the generic component) in the computation code of public methods. Components in ADLs are referred to as architectural units (AUs) (Figure 1c). An AU has in-ports and out-ports; ports are interaction points (or channels) for communication (by passing data/control) with the AU. The encapsulated component category (Figure 1d) includes EX-MAN components and web services; these components do not have the required interface. EX-MAN is an extended version of the X-MAN component model [23].

**Figure 2:**
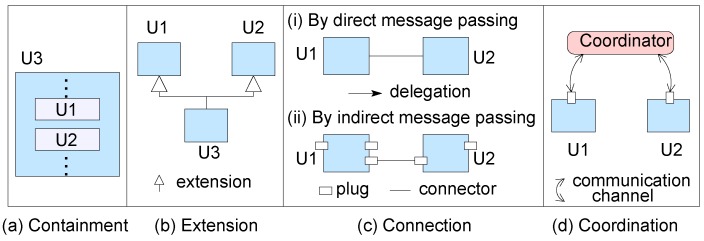
Four general categories of composition mechanisms.

In CBD, a component model defines a unit of programs, called component and composition mechanisms, to create bigger composite components from smaller components. The behavior and structure of this bigger unit is defined by the composing mechanism. A bigger system can be created by the composition mechanism if the mechanism is algebraic [24]; in other words, the composition of two or more components is another component of the same type to be composed further for system construction. In [15], these composition mechanisms are of four kinds (as shown in Figure 2): (i) containment, (ii) extension, (iii) connection and (iv) coordination.

In containment, to define a component, at least two components are put together for the composite. In extension, a composite is defined by extending the behavior from two existing components. A connection mechanism defines the interactions between two components; a connection can be either to pass messages directly or indirectly. In coordination, to create a composite of two components, a third coordinator program unit defines the coordination of control/data between the composed components.

## 3. Component-Based Incremental Construction Process

**Figure 3:**
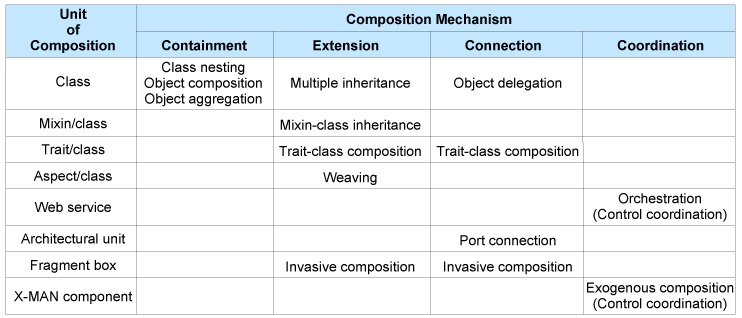
A taxonomy of software composition mechanisms.

Components in many component models are objects developed in an object-oriented programming language. For example, using Java, an EJB component can be created by using object aggregation/composition or inheritance mechanisms for objects. A COM component model supports a containment composition mechanism [25]. In the object-oriented development (OOD) paradigm, a class can be composed with aspects [26], mixins [27] and traits [28]. Hence, to complete our study of achieving incremental construction independent of any specific object-based component model, OOD itself is considered as a component model. Such a consideration is in compliance with the reference framework for the survey of component models defined in [29]. Using the four general categories of composition mechanisms, our proposed taxonomy (extracted from a survey on software composition mechanisms [15]) of software composition mechanisms in CBD is shown in Figure 3.

We conclude that the composition mechanisms from containment and extension categories, by and large, are used to define larger basic program units from the smaller program units. In contrast, composition mechanisms from connection (except trait–class composition) and coordination categories define interactions among the composed program units in the resultant composite unit; these mechanisms are useful to construct systems [30].

**Figure 4:**
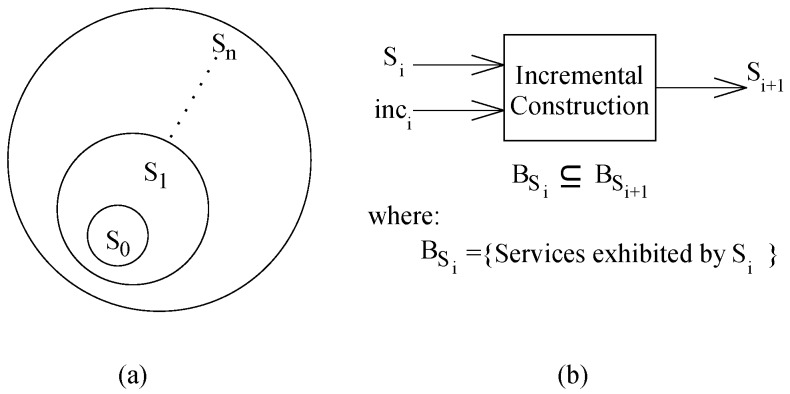
Incremental construction process.

In CBD, a system is a pair $\langle Comp,Comm\rangle$ of computation (components) and communication (connections or coordinators). Starting from $S_{0}$ (a component or a composite of components and connectors), incremental construction is a process (Figure 4a) to construct systems by adding increments (a component/connector/composite) to an incomplete system. The behavior of a system is based on both the computation and communication. It is a set of services exhibited by the system; a service’s role is the execution of computation from one or more components and possible interactions between the components. An incremental step (Figure 4b), relates the two consecutive systems as $B_{S_{i}}\subseteq B_{S_{i+1}}$. In this relation, $B_{S_{i}}$ and $B_{S_{i+1}}$ represent the behaviors of systems $S_{i}$ and $S_{i+1}$ respectively; the symbol ‘⊆’ represents the behavior containment, i.e., the functionality of the new system ($S_{i+1}$) contains the functionality of the old system ($S_{i}$). However, the exact structure of an increment and the precise nature of the behavior of the increment is different for different categories of component models.

**Figure 5:**
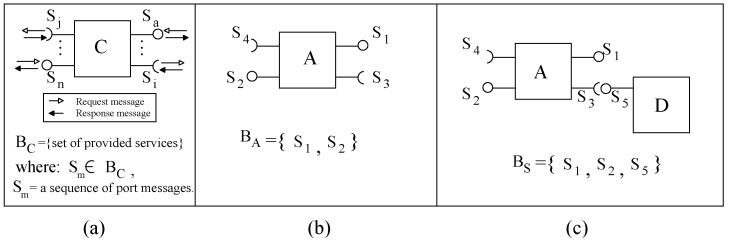
(**a**) Component definition, (**b**) Component behaviour, and (**c**) System behavior.

The concept of behavior containment is based on the functionality of components and system. Adding further details, the system pair ($\langle Comp,Comm\rangle$) can be represented as a tuple $\langle Comp,C,D\rangle$ of computation (Comp), control (*C*) and data (*D*). An execution of a system’s service may invoke computations from more than one component. Hence, for a service’s execution request, a system’s functionality is the *result* of executing its computations (according to its control flow) on its data. As with the system, the functionality of a component can be defined as a set of (provided) services exhibited by the component, e.g., component *C* in Figure 5a. The control flow in the computation of a service of a component defines interactions (tuple of $\langle Req,Res\rangle$ sending/receiving messages) on the component’s ports; this knowledge of interactions through ports is required to use the component in a system. Hence, the component’s functionality is a set of message sequences (Figure 5a). Interaction protocols in Wright [31], behavior protocol in SOFA2 [32], gate (port) protocols in TrustMe [33] and RDSEFFs (resource demanding service effect specification) in Palladio [34] component models use the concept of interactions on component ports.

In Figure 5b, we consider component *A* with two provided services $S_{1}$ and $S_{2}$ (with specific message sequences) and two required services ($S_{3}$ and $S_{4}$). As shown, service $S_{1}$ has dependency upon two external services $S_{3}$ and $S_{4}$ whereas service $S_{2}$ is independent of any external dependency. In a system, assuming that a provided port can be connected to more than one required port and a required port can be connected to one provided port, all provided and unconnected required component ports are ports of the system. A system by composing components *A* (initial system) and *D* (an increment with one provided service $S_{5}$) is shown in Figure 5c. The system’s functionality is a set of three provided services ($S_{1}$, $S_{2}$ and $S_{5}$); services on system ports (shown as $B_{S}^{\prime }$) or on system’s components’ ports (shown as $B_{S}$). The incremented system contains the behavior of the current system in two ways: (i) original services (e.g., $S_{2}$ of *A*) of the current system are exhibited by the new system and (ii) the incremented system offers new services (e.g., $S_{1}$ in Figure 5c) by combining services of the current system (e.g., $S_{1}$ of *A*) and of the increment (e.g., $S_{5}$ of *D*). The containment relationship between the two behaviors is expressed as ‘$B_{A}\subseteq B_{S}$’. In incremental construction, one advantage of this containment is that test cases of a current system $S_{i}$ can be useful for testing the incremented system $S_{i+1}$.

Out of many demanded features required for the construction of CPS, in this paper, we focus on achieving the feature of scalability by using our defined incremental construction approach. In this era of technology, an IoT-based CPS is constructed from a big set of requirements in steps. In a system construction method, with a support of reuseability in the construction step, smaller units are composed or constructed to make bigger units. The outcome of each such step should be a defined type for further reuse without losing the existing features of the composed units. In other terms, the composition is algebraic; this means the system construction can be achieved recursively. The incremental way of constructing systems would help us to construct systems of systems. IoT-based systems and cloud-based systems are two such examples of bigger systems constructed by composing existing conventional physical systems in order to work together. Keeping in view the complexity of these bigger systems, after each incremental step the outcome can be tested by using certain test cases. As no functionality is removed, test cases of previous partial systems can also be executed on the later partial and final systems; hence, the one direct benefit of our incremental construction process is the ability to automate the automatic testing of the intermediate as well as the final system. This feature of automatic testing is out of scope of this paper.

In truly CBD-based approaches, system construction is a bottom-up process, i.e., starting system construction from pre-built system-independent components [35,36]. For this study, we assume all components with functionality are available for system construction. In the rest of the paper, the behavior containment of the current system in the incremented system is investigated at the service level which can also be investigated at the messages level if necessary. In order to study the construction process in the three categories of component models, we consider a simple calculator example that evaluates a mathematical expression ($c=a^{2}+b^{3}$; where a, b and c are numeric variables). For the example in the study, we assume that each computational function is a provided service of a component (which may be calling other services in its code).

## 4. Incremental Construction in CBD

In this section, we investigate the current software component models with respect to the composition mechanisms defined in these models. The purpose of this investigation is to identify the most suitable component model for the construction of large scale systems incrementally. As mentioned in the previous section, current component models are of three kinds. Hence, we investigate these three kinds of models, and then based on a comparative analysis, identify EX-MAN as the suitable candidate for incremental construction.

### 4.1. Object-Based Component Models

In this section, we go through the composition mechanisms for objects (from Figure 3) to explore the possibility of using them to increment the behavior of a system. In this section, we also analyze the possibility of incrementing a system by adding code.

**Figure 6:**
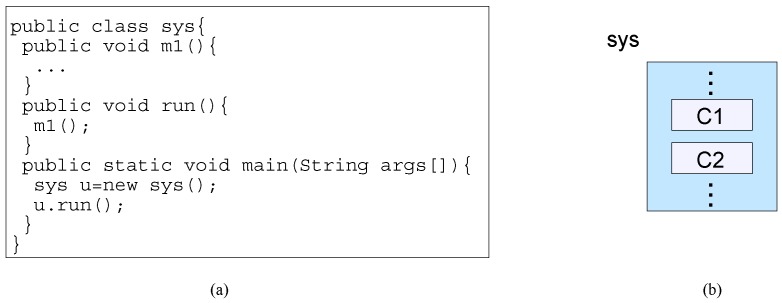
(**a**) Class nesting, object composition and object aggregation, (**b**) block diagram.

Objects/classes are composed by containment (class nesting, object composition and object aggregation), by extension (inheritance) and by connection (object delegation) mechanisms. For classes, containment and extension composition mechanisms construct a single class. In contrast, in OOD and CBD paradigms, a system is a composition of two or more objects connected by object delegation (message passing mechanism). The functionality of a single object (the *sys* class) is the execution result of the object’s *main* method, as shown in Figure 6a. The system’s behavior is to instantiate an object and to execute its *run* method.

For class nesting (from Figure 3), Java allows the code of inner classes (which are instantiated and used as normal objects) to be part of a class, as shown in Figure 6b. In order to achieve an increment in the system’s functionality by the composed inner classes, code must be added to instantiate the objects of the inner classes as data members of *sys* and then their methods must be called within the behavior of *sys*.

**Figure 7:**
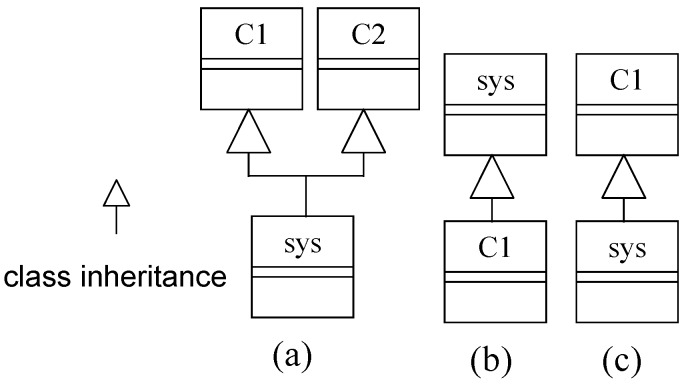
(**a**) Multiple inheritance, (**b**) Single inheritance, and (**c**) Single inheritance.

Similarly, *sys* can be incremented by multiple inheritance without overriding (Figure 7a) by the public members of the two parent classes C1 and C2. In invasive software composition [37], ‘single inheritance’ is also a composition mechanism because this composes two independently developed fragment boxes (class boxes corresponding to classes in OOD), as shown in Figure 7b,c. The inherited class is a pair of inherited part and incremented part [38]. Composition in Figure 7b is incremental if *C1* does not override any method of *sys*. As an alternate, composition in Figure 7c is incremental for not destroying (or overriding) the system’s behavior. However, the system functionality is not incremented by class inheritance alone; the extended member methods must be called from inside the system’s functionality.

**Figure 8:**
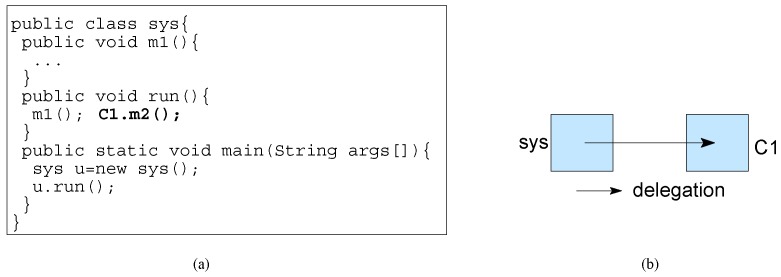
Object delegation in: (**a**) Code and (**b**) Block diagram.

Object delegation is a composition mechanism which composes two objects by one calling a method of another object. In Figure 8, we increment the functionality of the system by adding a method call (shown in bold) to object C1 inside the functionality of the system. To increment a system’s functionality, object delegation is an example of incremental construction if the method call is added in the behavior of the system.

**Figure 9:**
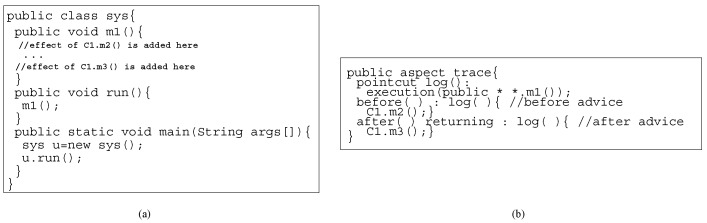
Aspect weaving: (**a**) Class, and (**b**) Aspect.

In aspects [26] (crosscutting concern), advices represent behavior that can be added at various join points. In Figure 9, we show how an aspect after weaving increments the behavior of *sys*. The aspect adds the effects of object delegations to object C1 in the method m1 of *sys*. To increment a system’s functionality, aspect weaving is an example of incremental construction if an aspect is weaved in the behavior of the system.

In mixin-class inheritance (from Figure 3) a mixin (an abstract subclass [27]) is composed with a class to increment the class. As with class inheritance, mixin-class inheritance without overriding adds functionality to the class but not to the system. Mixin-class inheritance with overriding overrides the functionality of the system; this mechanism is not behavior preserving and therefore not incremental.

**Figure 10:**
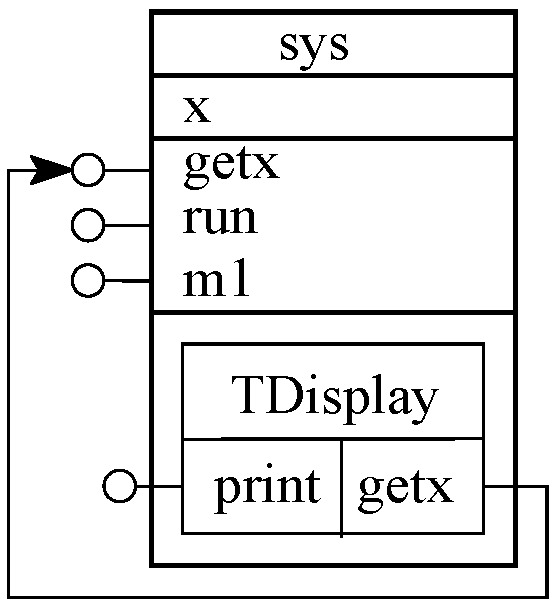
Trait-class composition.

Trait is a unit of reuse [28] which provides a set of services (shown with lollipop symbol and undertakes some useful computation) and may also require a set of services (shown with arrow symbol). In Figure 10, we show a composition of a TDisplay trait with *sys*. Composing a system class with traits by trait–class composition does not increment the functionality of the system. To increment the system’s functionality, the *print* method (newly added functionality to *sys*) must be called within the functionality of the system (e.g., the *main* method of *sys*).

**Figure 11:**
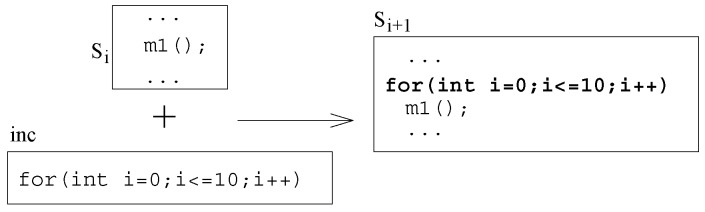
Increment by adding repetition construct.

We now show an increment based on code for a repetition construct, as shown in Figure 11. In the example shown, a repetition construct (for-loop) is added in the system behavior. In this increment, the functionality of the system is incremented by adding a loop to repeat calling a local method. Similarly, an increment to represent a select construct can be added to the system functionality to execute some behavior subject to the selection criteria.

**Figure 12:**
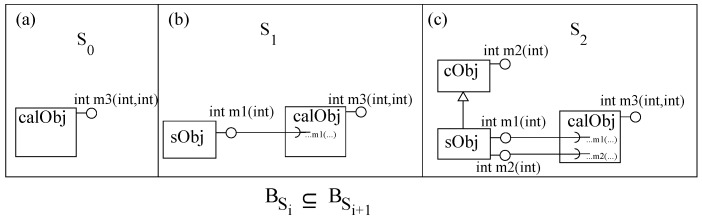
A system to evaluate a mathematical expression: (**a**) $S_{0}$, (**b**) $S_{1}$ and (**c**) $S_{2}$.

For the expression calculator example, we start with one component *calObj* ($S_{0}$) which has one method *m3* with minimum functionality (to add two numbers). In the next step, using aspect weaving, $inc_{0}$ *sObj* is added to the system by object delegation, as shown in Figure 12b. This increment adds a method call in *calObj* for a method of *sObj*. In the next step, we increment the system by inheritance (*sObj* has a new method from *cObj*) and add a local method call (again by aspect weaving) in *calObj*, as shown in Figure 12c. In both increments, the incremented system’s behavior contains the functionality of the system.

Another way of construction is to create a new coordination class *S* and construct the system by programming. In the system class *S*, first a call to method *m1* of *sObj* is made to get the squared value of a number. Next, a method call to *m2* of *cObj* is made to get the cubed value of a number. Lastly, a method call to method *m3* of *calObj* is made to get the addition of the squared and the cubed values. The system class *S* is coordinating with the three components.

### 4.2. Architecture Description Languages

In CBD, many component models are categorized as ADLs [29]. Composition in the mainstream ADLs do not change the internal computation code. Conversely, the invasive software composition (ISC) [37] changes the code of the composed components. For mainstream ADLs, components can have data, procedure (service) and/or event ports. A port connection can be of three types [39] a pipe (one-way data communication), a procedure-call (two-way message communication) and/or an event-broadcast (one-way message communication). The composition of AUs with three types of connectors creates three composition/architectural styles [40,41] (referred to as interaction styles in [42]): (i) pipe-and-filter style, (ii) client–server style and (iii) publish–subscribe style. A composition style with all three types of AUs and connectors is a hybrid style.

Component models support different architectural styles in different ways; for example, filter components in [43] have data (stream) ports and filter components in ProSave component model [44] have control ports. In this section, in order to avoid dissimilar features amongst component models with the same architectural style, we define three basic component models and analyze their support for incremental construction. The composition mechanism in these three component models is port connection and unconnected ports of the composed components are ports of the composite.

#### 4.2.1. The Basic Pipe-And-Filter Component Model

In CBD, for pipe-and-filter architectural style [41], a filter is an independent component with one or more input/output data ports; filter components read data from input ports, transform data and write data to the output ports. A pipe transfers data from the sender to the receiver ports. Pipe-and-filter architecture is formally defined in [43] and the ProSave component model (in the ProCom component model [45]) is based on a pipe-and-filter paradigm [44].

**Figure 13:**
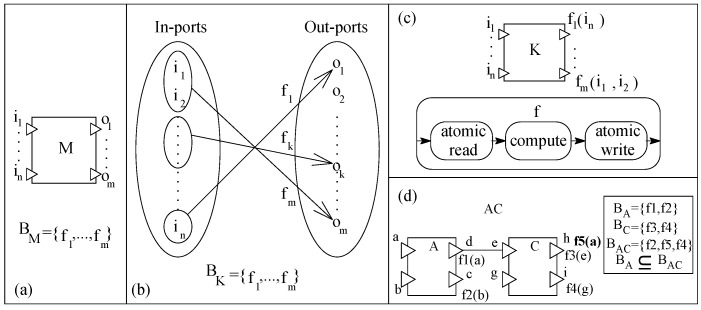
(**a**) A filter component, (**b**) Port relations, (**c**) Execution semantics, and (**d**) a composite.

In the basic pipe-and-filter component model (basic-pnf), a component is an AU with many in-/out-ports (Figure 13a). A component with at least one in-port/out-port is an eligible filter component in basic-pnf. Ports in filter components are channels for data communication. A data type is associated with each port that represents the type of data allowed to be communicated through the port. The behavior of a filter component is a set of functions and a function is a relation between a non-empty sub-set of in-ports and one out-port (Figure 13b shows the functionality of a specific component *K*). In order to keep the model simple, we assume that an in-port is related to one out-port. The functionality of a component can also be represented as a set of out-ports; where an out-port is simply a function of in-ports, as shown in Figure 13c for *K*. Figure 13c shows the execution semantics of a function of a component. In run-time, as soon as the data is available on in-ports, the related function is evaluated in three steps: (i) read in-ports atomically, (ii) compute the computation and (iii) write data on an out-port. Ports are read-destruct, which means that the data on ports will be destroyed once read.

In a basic-pnf, filter components are composed by port connections (referred to as a pipe to transfer between ports) between two non-similar (one in-port and one out-port) ports by matching the associated data types. The port of a filter component can be connected to one pipe. Composing two or more filters with pipes produces a composite filter (Figure 13d). All unconnected ports are the ports of the composite. In Figure 13d, initial system $S_{0}$ (component *A*) is incremented by connecting $inc_{0}$ (component *C*) through a pipe to create system $S_{1}$ (the composite *AC*). Function f5 (a compound function f1 of $S_{0}$ and f3 of $inc_{0}$) of *AC* relates the composite’s out-port ‘h’ with the composite’s in-port ‘a’. Corresponding with the definition of incremental construction from Section 3, f5 contains f1; hence, the relation of behavior containment between the two systems holds ($B_{A}\subseteq B_{AC}$). The basic-pnf component model supports incremental construction.

**Figure 14:**
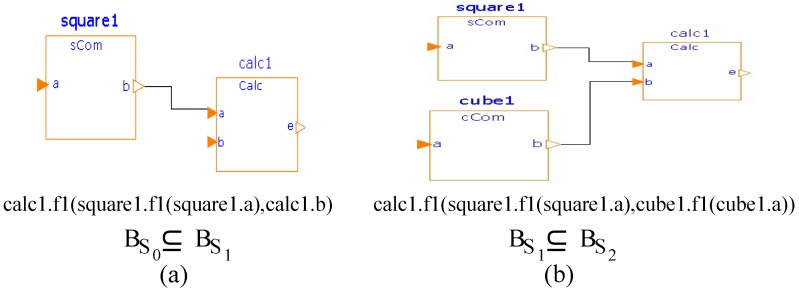
A basic-pnf System in Modelica: (**a**) First system and (**b**) Second system.

In order to illustrate incremental construction in basic-pnf, we use Modelica [46] to construct the system to evaluate a mathematical expression ($c=a^{2}+b^{3}$; where a, b and c are numeric variables). We develop three filter components (with one function each): (i) *calc* to add two integer values, (ii) *sCom* to square an integer value and (iii) *cCom* to cube an integer value. The system is constructed in two steps, as shown in Figure 14.

#### 4.2.2. The Basic Client–Server Component Model

**Figure 15:**
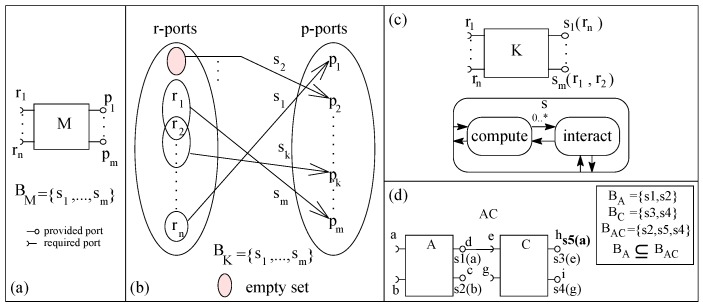
(**a**) A component, (**b**) Port relations, (**c**) Execution semantics, and (**d**) A composite.

Many component models (e.g., ACME, SOFA and UML) support the client–server composition style. In this style, two components communicate by passing messages through their ports. The execution of the caller service is paused by making a call and resumed on receiving the response from the called component. In our basic client–server component model (basic-cs), a component is an AU with many required ports (r-ports) and provided ports (p-ports), as shown in Figure 15a.

In basic-cs, a component with at least one p-port and zero or more r-ports is an eligible component. A port represents a message communication channel between a component and its environment. A signature (of a procedure/service) is associated with each port that represents the type of message allowed to be communicated through the port. A component’s functionality is a set of services and a service is a relation between a sub-set of r-ports and one p-port (Figure 15b shows the functionality of a specific component *K*). For a component, an interaction (sending a request message and receiving a response message) on an r-port is initiated from within the computation code of a service in the component. In basic-cs, an r-port of a component may be related to one or many p-ports and a p-port may be related to zero or more r-ports, as shown in Figure 15b. A p-port not related to any r-port represents that there is no external service request initiated within the computation associated with the p-port. The behavior of a component can also be represented as a set of p-ports; a p-port is simply a function of related r-ports, as shown in Figure 15c for *K*.

In basic-cs, during execution, only one service of a component may be executed at a time. Figure 15c shows the execution semantics of a service of a component; a service interacts zero or more times on its r-ports. The execution of a service with associated r-ports is paused by requesting external services on its r-ports. The paused service resumes its execution after receiving a response message (control/data) from the requested service. In basic-cs, components are composed by port connection between ports with matching associated service signatures. For simplicity, we consider only one connection for a port. A composite of two components is shown in Figure 15d; all unconnected ports are composite’s ports and the behavior of the composite is a set of services (or unconnected p-ports).

In Figure 15d, an initial system $S_{0}$ (component *A*) is incremented $inc_{0}$ (component *C*) by port connection and the composite *AC* is the incremented system $S_{1}$. A compound service s5 relates the composite’s p-port ‘h’ with the composite’s r-port ‘a’. As the relation of behavior containment between the two systems holds ($B_{A}\subseteq B_{AC}$), basic-cs component model supports incremental construction.

**Figure 16:**
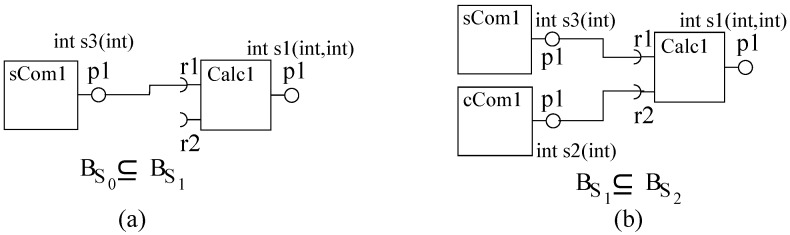
A basic-cs system in ArchJava: (**a**) First system and (**b**) Second System.

In order to illustrate incremental construction, we consider constructing a system to evaluate the mathematical expression ($c=a^{2}+b^{3}$). We develop three components *Calc* (to add two numbers), *sCom* (to square a number) and *cCom* (to cube a number) in ArchJava [47,48]; the desired system is constructed in two steps, as shown in Figure 16.

#### 4.2.3. The Basic Publish–Subscribe Component Model

In the publish–subscribe architectural style, messages flow from one publisher component to many subscriber components. The publish–subscribe style is suitable to construct a system where many different components have to perform their specific computations subject to an event in one common component. In CBD, many component models (e.g., ACME, C2 [49] and SOFA) support this style.

**Figure 17:**
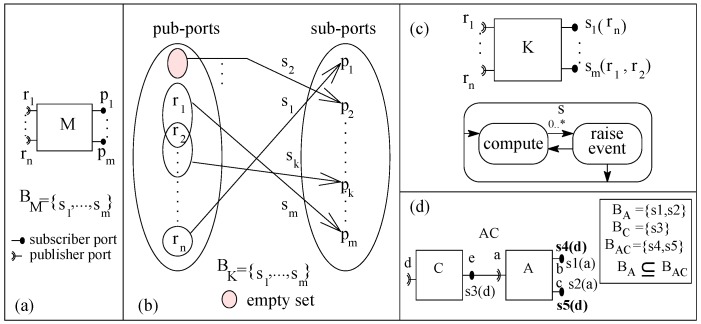
(**a**) A component, (**b**) Port relations, (**c**) Execution semantics, and (**d**) A composite.

In the basic publish–subscribe (basic-ps) component model, a component is an AU with zero or more publisher ports (pub-ports) and with one or more subscriber ports (sub-ports) (Figure 17a). A message signature is associated with each port that represents the type of message allowed to be communicated through the port. The functionality of a component is a set of services and a service is a relation between a subset of pub-ports and one sub-port (Figure 17b shows the functionality of a specific component *K*). A sub-port only allows a message to pass into the component and a pub-port only allows a message to go out of the component. In basic-ps, for a component, one pub-port may be related to many sub-ports, as shown in Figure 17b for *K*. A component may have services in which no event is raised within the service. The behavior of a component can also be represented as a set of sub-ports; sub-ports are simply functions of pub-ports, as shown in Figure 17c for *K*. Components in basic-ps have their own control. Figure 17c shows the execution semantics of a service of a component. A service executes by receiving notification on the associated sub-port and raises event(s) on associated pub-port(s).

In basic-ps, components are composed by port connection between matching ports, as shown in Figure 17d; the result of composition is a composite. In basic-ps, for simplicity, we consider that a sub-port may be connected to one pub-port at a time and a pub-port may be connected to zero or more sub-ports. All unconnected ports of the composed components are the respective ports of the composite. In Figure 17d, considering component *A* as initial system $S_{0}$ and component *C* as increment $inc_{0}$. Composite *AC* is the incremented system $S_{1}$ and service s5 of $S_{1}$ contains s2 and s3. As the relation of behavior containment between the two systems holds ($B_{A}\subseteq B_{AC}$), basic-ps component model supports incremental construction.

**Figure 18:**
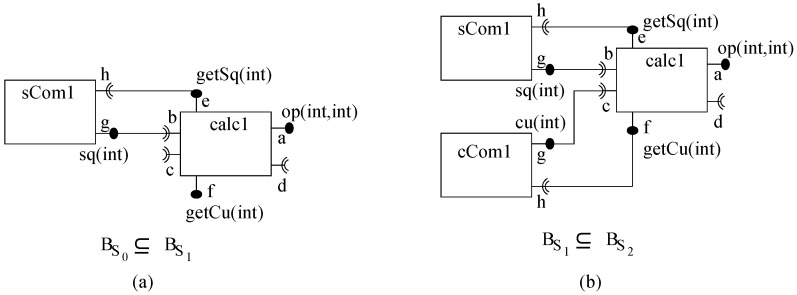
A basic-ps component model system in ArchJava: (**a**) First system and (**b**) Second System.

To illustrate incremental construction, a system to evaluate a mathematical expression ($c=a^{2}+b^{3}$) is constructed from three components *calc*, *sCom* and *cCom* in ArchJava as shown in Figure 18. The *op* service of *calc* accepts two numbers (of type integer) as arguments and broadcasts on its ports ‘b’ and ‘c’. Service *getSq* receives the result on port ‘e’ and raises an event on port ‘d’ if port ‘f’ has been notified. Service *getCu* receives result on port ‘f’ and raises an event on port ‘d’ if port ‘e’ has been notified. Component *sCom* offers service *sq* to square a number and to broadcast the result on port ‘h’. Similarly a component *cCom* offers service *cu* to cube a number and to broadcast the result on port ‘h’.

#### 4.2.4. Special Cases of Incremental Construction

**Figure 19:**
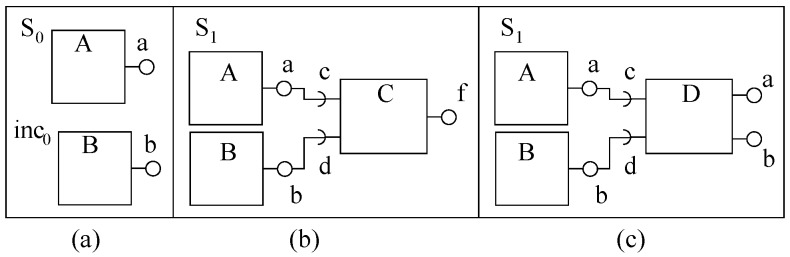
Components with provided ports only: (**a**) Components, (**b**) First system, and (**c**) Second system.

For constructing a system by using ADLs, the only way to add an increment is by port connection. Practically, we may face situations when an increment component cannot be connected to the current system. Considering generic AUs, we discuss two such cases shown in Figure 19 in this section.

In the first case, considering any two components *A* (as $S_{0}$) and *B* (as $inc_{0}$) with generic provided ports only (Figure 19a), a special component is needed just to compose these two components in two possible ways. The component *C* (Figure 19b) can coordinate communications between components *A* and *B*. Similarly, just to compose, a component can be created to forward the ports of the two components, as shown in Figure 19c. In order to compose such components, the ProCom component model introduces special AUs (called connectors) to coordinate components [44].

**Figure 20:**
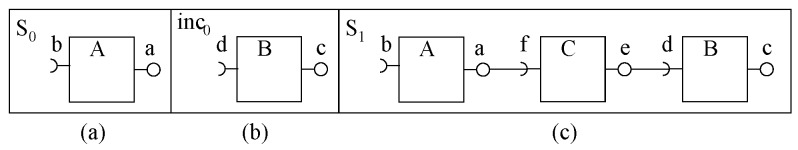
Components with incompatible ports: (a) Component, (b) Increment, and (c) System.

In the second case, as shown in Figure 20, we consider two components *A* and *B* with no matching ports. In order to compose such components, an adapter component is needed that does not have any functional behavior in the system. Components with incompatible ports in [50] are connected by using adapting filters; these filters adapt the type of message from one port to the acceptable type by the other port.

#### 4.2.5. Invasive ADL

**Figure 21:**
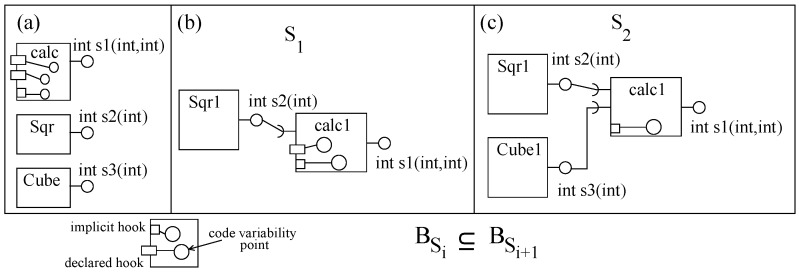
A system of invasive ADL components: (**a**) Components, (**b**) First system, and (**c**) Second system.

Invasive software composition (ISC) [37] uses the concept of aspect weaving (described in Section 4.1) to change the code of a component. Hence, this component model is different than the mainstream ADLs. This model uses another different way to increment a system and that is by transforming the component code to extend and to connect with other components. For incremental construction of the calculator, we consider three components with provided ports only (Figure 21a).

Component *calc* has two declared and one implicit hook. In the first incremental step, component *calc1* ($S_{0}$) is composed with component *Sqr1* ($inc_{0}$) by using a composer program *comp1* (not shown). Composer *comp1* invades into one of the declared hooks to extend and connect *calc1* with *Sqr1*, as shown in Figure 21b. In the next incremental step, the system from Figure 21b ($S_{1}$) is composed with Cube1 by using another composer program *comp2*. Composer *comp2* invades into *calc1*’s declared hook to extend and connect *calc1* from ($S_{1}$) with *Cube1* (Figure 21c).

Through the composer program, the declared hooks from a component’s composition interface are disappeared. However, the implicit hooks are still available in the component interface for further transformations. In the example used, the transformed functionality of *calc1* is contained by the three connected components. After each incremental step, the behavior of the incremented system ($B_{S_{i+1}}$) contains the functionality of the previous system ($B_{S_{i}}$). Using this component model, components with incompatible ports are not required to be composed by a third component (as shown in Figure 20), but such components can be transformed by a composer program for connection.

### 4.3. Component Models with Encapsulated Components

From the current component models, we have included two component models in which components have provided ports (interfaces) only; such components are referred to as encapsulated components. In this section, we briefly investigate system construction with web services and with encapsulated components in EX-MAN. As there are no dependencies in this category of components, component model (web services, X-MAN and EX-MAN) with these components are more suitable than other two categories.

### 4.4. Web Services

Web services can be composed by either using orchestration mechanism or by using choreography mechanism [51,52]. A composite web service using the choreography mechanism will not be an encapsulated component. In contrast, a composite web service using orchestration mechanism is an encapsulated component. As orchestration is a more common mechanism, we consider this mechanism in this paper for making the composite service an encapsulated component.

**Figure 22:**
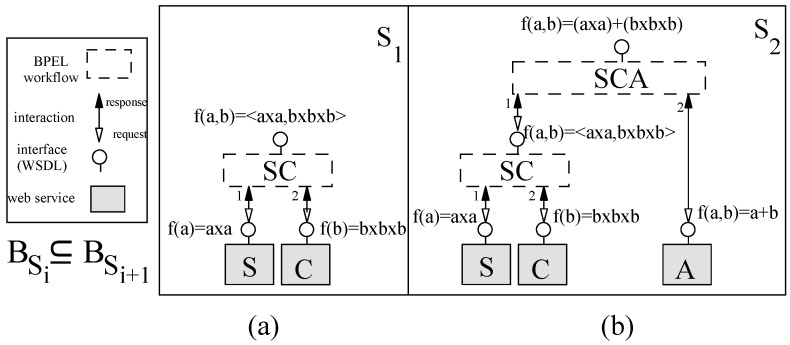
Web services: (**a**) First system, and (**b**) Second system.

The behavior of a web service is a set of its operations specified in its web service description language (WSDL); web services are composed by programming a BPEL process which coordinates control between the composed web services [52]. The BPEL process can then be converted to a web service for further composition. To illustrate incremental construction by web services, we construct the calculator as shown on Figure 22.

For constructing the calculator example, we start by composing a web service *S* ($S_{0}$; with one operation) with another web service *C* ($inc_{0}$) by a BPEL process; the BPEL process is then converted to a web service *SC* ($S_{1}$ shown in Figure 22a). This composite has one operation which accepts two numbers and returns a pair. In the second incremental step (Figure 22b), *SC* is composed with web service *A* ($inc_{1}$) by a BPEL process; the BPEL process passes the values returned by *SC* as input to *A*. The BPEL process is then converted to a web service *SCA* ($S_{2}$). In Figure 22, numeric labels next to the interaction arrows represent the order of their occurrence. After each incremental step, the functionality of the incremented system ($B_{S_{i+1}}$) contains the behavior of the previous system ($B_{S_{i}}$).

### 4.5. EX-MAN Component Model

The conventional and fundamental X-MAN component model was defined with encapsulated components and exogenous connectors [53,54]. Pre-defined exogenous connectors [55] is a unique set of connectors which play a vital role for the suitability of X-MAN for incremental system construction. The concept of exogenous connectors are also proposed to compose web services [56]. However, being an abstract component model, X-MAN does not define many features precisely. For this reason, there are many different definitions of some exogenous connectors and these are implemented in many different ways in the supporting tools (used in [57,58,59,60,61,62]) of X-MAN which is not re-producible. In order to overcome these limitations of X-MAN, without violating the fundamental concepts of the model, we extended the model by addressing the limitations of X-MAN; this extended model is referred as EX-MAN [11,12,63]. With regards to the strongest feature of X-MAN, the exogenous connectors are precisely defined to be functioning with fixed behavior with the help of flow constraint language (FCL) [63] defined in EX-MAN.

**Figure 23:**
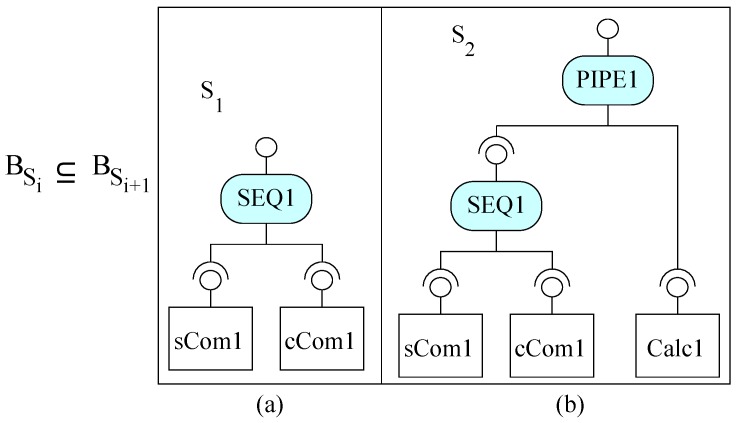
EX-MAN: (**a**) First system, and (**b**) Second system.

In EX-MAN, components have one provided interface and components do not call other components’ services directly. Components are composed by exogenous composition connectors. The functionality of a component is the set of services exhibited by its interface. To construct the calculator example, we assume three atomic components *Calc* (with a service to add two numbers), *sCom* (with a service to square a number) and *cCom* (with a service to cube a number). By using instances of these components, we compose the system in two constructional steps, as shown in Figure 23. In the first step, we compose *sCom1* ($S_{0}$) with *cCom1* ($inc_{0}$) by a sequencer connector (SEQ1). In composite $S_{1}$ (Figure 23a) SEQ1 executes a service from *sCom* and then a service from *cCom*. In the second step, we add *Calc1* ($inc_{1}$) with the current system $S_{1}$ by means of a pipe connector (PIPE1). In this composite $S_{2}$ (Figure 23b), the computed results from SEQ1 are passed as input values to *Calc1* by PIPE1. After each incremental step, the functionality of the incremented system ($B_{S_{i+1}}$) contains the behavior of the previous system ($B_{S_{i}}$). To avoid cluttering, we only shown the minimum feature of the EX-MAN system which is necessary for this study.

### 4.6. A Comparative Study of the Three Categories of Component Models

**Figure 24:**
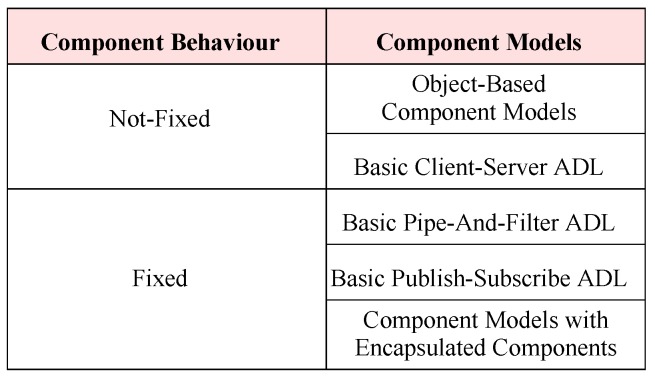
Behavior-based categories.

With respect to component behavior, we categorize the current component models from the three categories into two groups, as shown in Figure 24. Using component models from the category of not-fixed behavior for incremental construction, a system’s functionality may be fixed once the system is completed. In contrast, using component models from the category of fixed functionality for incremental construction, the final system as well as the intermediate systems have fixed functionality; the functional behavior of such systems can be verified.

**Figure 25:**
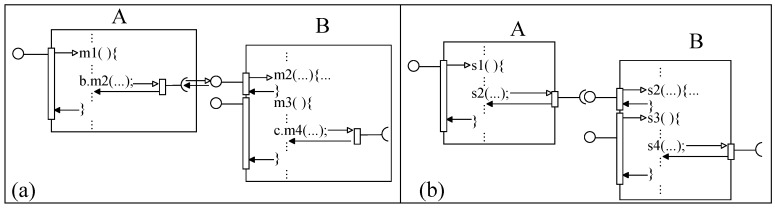
Behavior of Components: (**a**) First view, and (**b**) Second view.

In a composite of two components *A* and *B* (Figure 25), component interaction begins/ends on a provided port with a request/response message, as described in Section 3. In general, objects have external method calls in their methods; an object with external method calls (Figure 25a) does not have fixed functionality with respect to control and computation. With respect to control and computation, the functionality of *A* and *B* are not fixed as including one of these components implies to have more components; hence, this leads the developer to add more components. In object-based component models (from Section 4.1), a truly component-based construction may be achieved artificially if components are implemented as encapsulated components, as shown in Figure 12. However, achieving incremental construction by the weaving mechanism is limited as the code (e.g., external method calls) cannot be inserted everywhere in a component to achieve behavioral increment to the system.

In basic-cs (from Section 4.2.2), a typical AU has required ports; hence, functionality of an AU is not-fixed with respect to control and computation. Required ports of a component represent the dependencies of the component upon other components with matching ports. As with object specific composites, the functionality of component *A* (Figure 25b) depends on the functionality of some component (e.g., *B*) compatible to be connected with component *A*. Computation of component *A*’s method or service halts by making request to a method or service of component *B*; *A*’s halted computation resumes after receiving response from *B*. In contrast, AUs in basic-pnf and basic-ps styles have their own control. For a filter component, a computation is fixed to read data from the in-ports and to produce data on the out-ports. Similarly, in the publish–subscribe style, computation of a component is fixed to listen to event notifications on the sub-ports (subscriber ports) and to raise events on the pub-ports (publisher ports). Component for these two styles have fixed behavior.

For not having required ports, component models with encapsulated components are placed in the category of fixed behavior. In some component models, increments can also be added by programming (or by refactoring) and by adapting. Another way to increment an existing system seems to be by substituting an existing component with another component (with the signature based compatible ports). However, substituting a component by another component with the compatible ports may raise port tracing issue (sequence of messages of provided services) [33]. Moreover, non-functional properties of the services offered by the two components (developed by different vendors) may be different.

## 5. System Construction in EX-MAN

**Figure 26:**
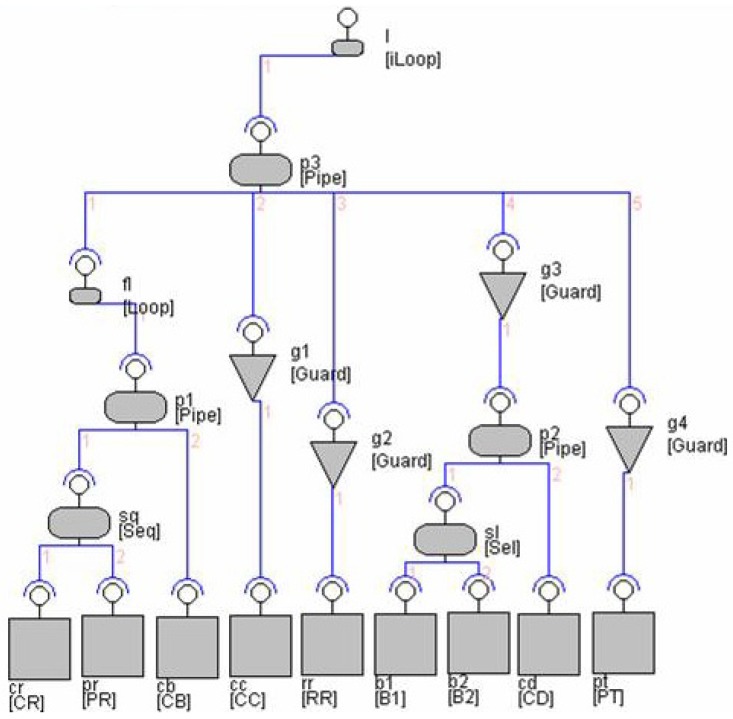
The ATM system.

**Figure 27:**
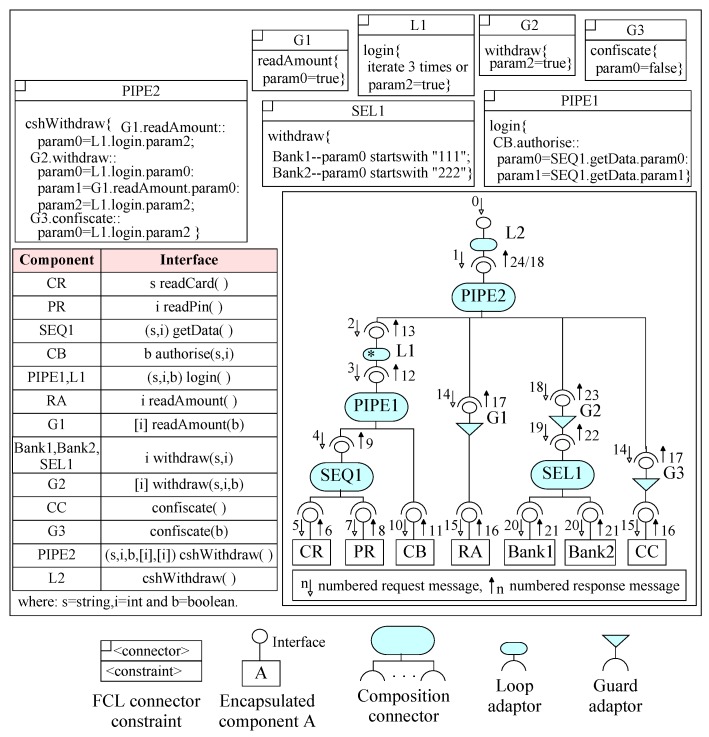
The ATM system detailed design.

Using the defined process of incremental construction in Section 3, we have modeled and simulated a number of EX-MAN systems in a tool called Exogenous Composition Framework (ECF). In this section, we show an ATM system example with full details and another two system designs without details. The details of these systems can be found in [11,12]. The ATM system constructed in the ECF tool of EX-MAN is shown in Figure 26. The details of this system in shown in Figure 27.

### 5.1. ATM System

Using EX-MAN for incremental construction, Figure 26 shows the design of an ATM system in the ECF tool of EX-MAN. The system is constructed incrementally by following our defined approach. The final system as well as the intermediate partial systems during the incremental construction process were tested for the added and preserved behavior.

A system in EX-MAN (shown in Figure 27) is comprised of components to perform computational tasks (in the bottom layer of the architecture) and connectors to perform control/data coordination (in multiple layers). The EX-MAN design of the ATM system includes the details of component interfaces, connector’s flow constraints (written in flow constraint language (FCL) [63] for EX-MAN) and overall request/response flow with arrows in the system.

A component interface is a collection of services. In the ATM system, each component has a single service. A connector in the architecture can be either a composition connector (sequencer SEQ, pipe PIPE and selector SEL) or an adaptor (guard and finite/infinite loop). In the system architecture, an interface appearing on a connector represents a composite component (if on a composition connector) or an adapted component (if on the adaptor connector); hence, components with the respective interface are shown including the encapsulated components as well as the composite/adapted components.

On installation of the ATM system, request annotated with ‘0’ is initiated to the root loop connector L2; L2 is an infinite loop connector which passes the request to the adapted system (PIPE2). On a service request, a PIPE connector makes a request to each connected component in order from left to right. This connector is enabled to pass the results of a service from a component as input arguments in a service request to other components. These details of passing results are defined in a FCL constraint for the PIPE. PIPE2 makes the first request to the finite (marked with ‘*’) loop connector L1. L1 is constrained to repeat a request until the true result is produced in maximum three iterations by the adapted component. From L1 to PIPE1 and then to sequencer SEQ1. A sequencer is similar to pipe with the ability to pass the results of a service as arguments to another service. SEQ1 makes a request to component CR to read an ATM card number and then a request to PR to read pin number; these two numbers are passed to component CB in the request by PIPE1. If the card is authenticated by component CB then L1 terminate its iterations and returns the response to PIPE2. PIPE2 then passes the request to next component; after checking the authentication by guard G1, the request is sent to component RA to get a transaction details from the system user. The next request by PIPE2 is checked by guard G2 for card authentication and passes the request to one of two bank components. In case the card authentication failed, the last request by PIPE2 (after being checked by guard G3) is passed to component CC to confiscate the card.

### 5.2. Weather Information System

A weather information system case study from [7] is linked with many remotely located weather stations. A weather station has a system linked with a number of sensors and devices to read different values from the weather. These values are collected through the sensors automatically and a number of times in a day. The central weather information system collects the data from these weather stations through a satellite communication link. In this section, using our approach, we construct a system for weather information system to get data from two remote weather stations.

First of all, using EX-MAN, we prepared a repository of nine basic components (ground temperature (GT), air temperature (AT), air pressure (AP), wind speed (WiSp), rain fall (RF), wind direction (WD), humidity (HM), store data (DS) and component (SC)) required for the construction of considered case study. A component to read the ground temperature (GT) is created. This component is capable to compute minimum, maximum and average temperature values from the collected data from any specific period. Similarly, in the repository, another component to read the air temperature (AT) is created; this component provides minimum, maximum and average temperature values form the collected data form any specific period. Another component to compute the minimum, maximum and average values for the air pressure (AP) is created. For measuring the minimum, maximum and average values for the wind speed (WiSp) is created. A component to measure rain fall (RF) and a component to get the wind direction (WD) are created. To measure minimum, maximum and average values for humidity (HM) is created. A component to store data (DS) is created to save data from components connected to sensors. Lastly, a component (SC) is created to establish the satellite link with the remote weather station.

**Figure 28:**
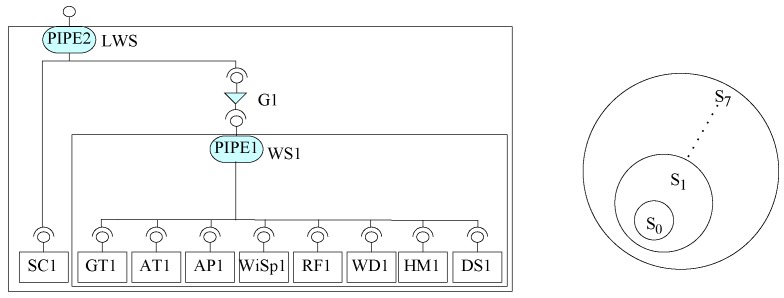
Construction of linked weather station (LWS) composite component.

In order to create the desired system by using our approach, we constructed a composite component linked weather station (LWS) for the linked weather station. For this purpose, the nine basic components created are used to construct the composite LWS in eight steps from $S_{0}$ to $S_{7}$, as shown in Figure 28. In the construction step, from the repository components, component instances are created for composite construction. In the first step, two component instances GT1 and AT1 are composed with a PIPE instance PIPE1; this composite is referred to as partial system $S_{0}$. In the second iteration, $S_{0}$ is incremented by an instance of AP1; this composite is referred to as partial system $S_{1}$. Next, $S_{1}$ is incremented with component WiSpi to create $S_{2}$. In the next incremental step, partial system $S_{2}$ is incremented with component RF1 to create partial system $S_{3}$. Similarly, for the next step, partial system $S_{3}$ is incremented with component WD1 to produce partial system $S_{4}$ and $S_{4}$ is incremented with the component HM1 for the creation of partial system $S_{5}$. Next, partial system $S_{5}$ is incremented with DS1 component to create partial system $S_{6}$. In the last incremental step, partial system $S_{6}$ is incremented with a component SC1 and two connectors PIPE2 and guard G1 to create composite component $S_{7}$. This component is saved in the repository as component LWS.

**Figure 29:**
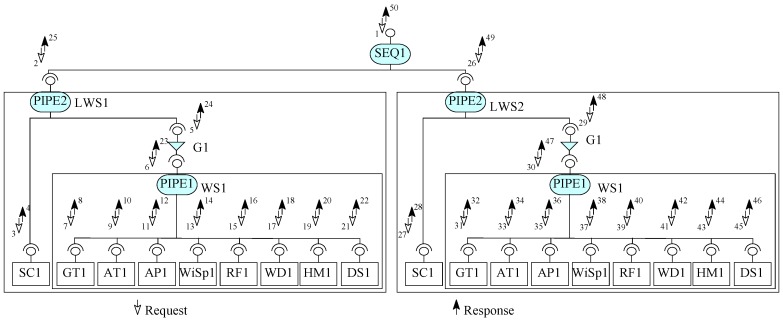
Two linked weather stations (LWS) in the Weather Information System.

Next, we have constructed a prototype for a weather information system linked with two remote wilderness weather stations (shown in Figure 29). For this construction, in the first step, an instance of composite component (shown in Figure 28) LWS1 is created and is composed with another instance of the same composite component LWS2 by using a sequencer connector instance SEQ1. In order to get the data from these two linked weather stations, a request will be made through the SEQ1 connector and a response containing the data from both weather stations will be generated by SEQ1.

In Figure 28, the internal construction of the two weather stations LWS1 and LWS2 are shown with request/response arrows to depict the flow of data and control through the system. To get weather data from two weather stations, a request (numbered 1) is made through the SEQ1 connector. This connector splits the received request into sub-requests (one for each composed component) to LWS1 and LWS2. Once the response of the first request to the first composed component LWS1 is received then the next sub-request is made to next composed component. This sequence is also shown with the help of numbered arrows.

The request to the first LWS1 is received by PIPE2 connector. This connector is basically a sequencer with an added feature of passing data from the response of one component into the request of later components. PIPE2 sends the request to SC1 to establish the satellite link and pass the response of this request (successful or unsuccessful) from SC1 to guard connector G1. If the result from SC1 is successful then the G1 allows the request to go ahead to the WS1 component. PIPE1 in WS1 makes seven requests in sequence to the connected components from component GT1 to component HM1. For each request, the respective component returns the observed and computed data to PIPE2 as a response. After getting data from these seven components, PIPE1 passes the collected data from seven components as request to component DS1 to store the data locally. Finally, PIPE1 prepares a response for the received request from G1 which is then sent to PIPE2 and then to SEQ1 as the final response from component LWS1. Similarly, SEQ1 makes the request to the second component LWS2 and collects the response and then prepares a response numbered as 50 for the request numbered 1.

### 5.3. Cash Desk System

**Figure 30:**
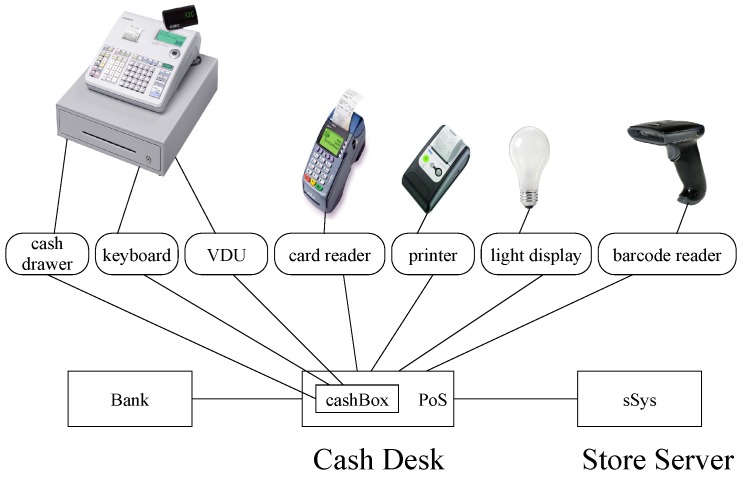
The cash desk system of common component modeling (CoCoME).

Using our approach for constructing systems in EX-MAN, we have developed a complex system using the common component modeling (CoCoME) defined in [64]; this system is a benchmark in CBD. The cash desk sub-system (linked with many devices as shown in Figure 30) of CoCoME was modeled and simulated in ECF.

The cash desk system of CoCoME is a typical point-of-sale (PoS) system operating in department stores; this system is connected with many devices as shown in Figure 30. However, the system may be installed with a shopping cart in the future to implement the concept of a smart shopping cart. In the conventional set up, a customer comes to the cash desk to get the shopping items processed and to pay the bill for check out. The human attendant operating the system scans each item to get its price and other details from the system. After scanning all items the customer is given the option to pay either by a bank card or by cash. The cashbox component communicates with three peripherals (cash drawer, keyboard and VDU). The barcode reader is used to scan item IDs one by one; a barcode can also be entered from the keyboard if the barcode reader is unable to scan the code correctly. The VDU displays a list of scanned items with their prices. In order to accept payments from a customer’s card (read by the card reader device), the PoS system communicates to the bank system. Cash and receipts of card payments are kept in the cash drawer. The printer is used to print receipts for purchased items and card payments. With the display of a colored light bulb, the system either runs in normal mode or in express checkout mode. In express checkout mode, customers are restricted to checking out at most eight products, and the card payment is prohibited.

**Figure 31:**
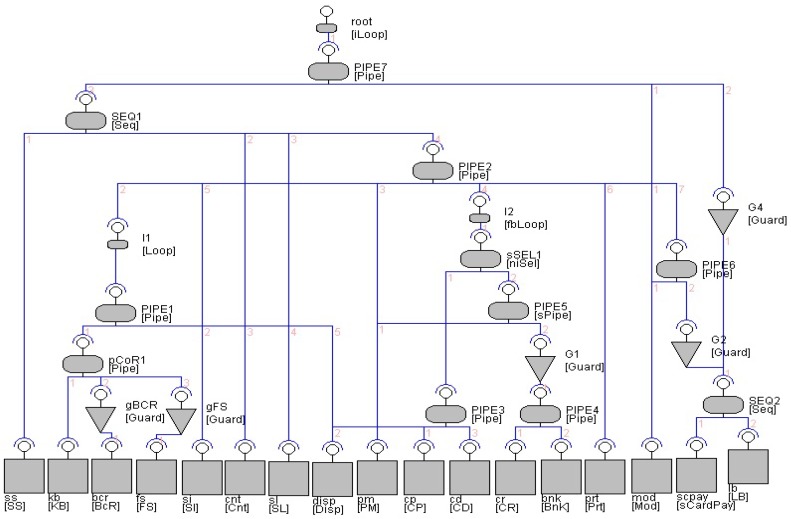
EX-MAN design of cash desk sub-system in Exogenous Composition Framework (ECF).

The EX-MAN design of the cash desk system with the ECF tool is shown in Figure 31. The complete system is created in 26 incremental steps. Initially, component SS (to start sale) is selected from the repository to make $S_{0}$ and then in the next step component KB (to allow bar code entry by keyboard) is selected to increment the system $S_{1}$. Similarly, in 24 incremental steps the complete system is constructed. After adding each increment, the system was tested for the previous system behavior and for the incremented system behavior. To avoid cluttering, the request and response arrows are not shown in Figure 31. The complete and detailed operational working of the system is given in [11].

## 6. Discussion

With the advancements in the sensor technology, IoT-based systems are constructed by integrating hardware and software components together. Incremental construction defined in this paper is suitable for systems developments in a domain in which a system’s requirements do not change quite often, e.g., the automotive domain in which features/specification of future products/systems is fixed. So which component model should we choose for incremental construction? The intention is to pick a component model which can help constructing a system incrementally (in many iterations) such that the system has fixed functionality after each increment. The benefit of a system with fixed behavior is that the system’s functionality is verifiable after each increment without stubs (artificial components producing expected result values without real computation). Being able to verify a system with fixed functionality reduces the extra overhead of stubs for sub-components [65].

With the defined incremental construction process (from Section 3), we investigated the possibility of using current component models to address the scalability issue of bigger and complex systems. In regard to the fixed or not-fixed behavior of a system, we comparatively analyzed (in Section 4.6) the current component models and found encapsulated components as the most appropriate choice to achieve the scalability issue.

Encapsulated components are defined in X-MAN, EX-MAN and service oriented architecture (SOA [66,67]). In the absence of dependability, all these three approaches inherently support incremental system construction. EX-MAN is an extended version of X-MAN with many advantages. For the composition of encapsulated components in SOA, there are no pre-defined composition programs of connectors; to compose existing services, orchestration is the common practice by using BPEL. In contrast, for the composition of encapsulated components in X-MAN and in EX-MAN, pre-defined program units called exogenous connectors are available. In contrast to exogenous connectors in X-MAN, connectors in EX-MAN are annotated with specifications written in a flow constraint language to fix the data/control flow for composition [63]. Hence, in this paper, using EX-MAN, we demonstrated the construction of two toy systems (calculator and ATM), one large and complex cash desk system and a CPS weather information system.

## 7. Conclusions and Future Work

To have a system with fixed functionality by using a component model from the ‘Not-Fixed’ category, the construction process may begin with a component without any dependencies (e.g., an encapsulated component). Then an increment is added to the system to create the incremented system such that the system has fixed functionality. In the worst case, the system may be constructed in a single step (big-bang integration) if there is no encapsulated component for constructing a system. In contrast, component models from the other (fixed functionality) category can be used to construct the system in the desired way. Pipe-and-filter style systems are data driven systems. A component model with publish–subscribe composition style is more appropriate for building graphical user interface (GUI) software [49]. In both styles, a system has many connections (one way flows of data/control) between the components. Systems constructed by with web-services or by EX-MAN have tree structure architecture and the composition mechanism is control coordination (Figure 3). In contrary to the composition (an arbitrary BPEL process) in web-services, the composition (by pre-defined exogenous connectors) produces an encapsulated component to be further composed; hence, behavior containment is automatically preserved in EX-MAN. Moreover, in EX-MAN systems, computation (within components) and control (within connectors) can be clearly segregated; this may not be possible in a system constructed by web services as the BPEL processes can also have some computation code [65].

There are many methodologies to address the issue of constructing large software systems in steps. In a step, a functionality may be added, amended or deleted. The unique advantage of the proposed approach is the addition of newer functionality in each step. For the construction of system in which the change is minimum, the proposed approach can be applied. After adding each step the system may be tested automatically. With the defined approach, the selected EX-MAN component model is used for the fixed pre-defined connectors. Such a component model makes the use of incremental construction process easier.

With a special emphasis on the behavior containment, this paper contributes a redefinition of the notion of incremental construction for the construction of large and complex systems. Next, we contribute a study of applying the defined approach on current component models to investigate how easily large systems can be constructed in CBD. By studying and comparing the strengths and weaknesses of current component models, we propose a taxonomy of component models with respect to component/system behavior and select the EX-MAN component model (EX-MAN) for its comparative suitability for incremental construction. For the evaluation of our scalable approach, in this paper, we discuss three different systems in EX-MAN.

In conclusion to our study of achieving incremental in the current component models, we find EX-MAN the most appropriate model for the construction of systems incrementally. However, the majority of the component models in CBD are ADLs; for future work, we would like to work on an ADL with the property to achieve incremental construction with ease. The automatic testing benefit of our defined approach (discussed in Section 3) can be worked on in our future endeavors for the continuity of this research.
